# Features of cough variant asthma and classic asthma during methacholine-induced brochoconstriction: a cross-sectional study

**DOI:** 10.1186/1745-9974-5-3

**Published:** 2009-03-09

**Authors:** Hisako Matsumoto, Akio Niimi, Masaya Takemura, Tetsuya Ueda, Masafumi Yamaguchi, Hirofumi Matsuoka, Makiko Jinnai, Kazuo Chin, Michiaki Mishima

**Affiliations:** 1 Department of Respiratory Medicine, Kyoto University, Kyoto, Japan; 2 Department of Respiratory Medicine, Kitano Hospital, Osaka, Japan; 3 Department of Respiratory Care and Sleep Control Medicine, Kyoto University, Kyoto, Japan

## Abstract

**Background:**

Little is known regarding mechanistic and phenotypic differences between cough variant asthma (CVA), presenting with a chronic cough as the sole symptom that responds to bronchodilators, and classic asthma with wheezing during methacholine inhalation. Here we reported airway sensitivity, airway reactivity, and as the main concern, the appearance of cough and wheezes during methacholine inhalation in patients with CVA or classic asthma.

**Methods:**

We cross-sectionally examined the degrees of airway sensitivity, the point where resistance started to increase, and reactivity, the slope of the methacholine-resistance curve, and the appearance of cough and wheezes in steroid-naïve adult patients with classic asthma (n = 58) or CVA (n = 55) while they were continuously inhaling methacholine during simultaneous measurement of respiratory resistance.

**Results:**

Patients with CVA were less sensitive and less reactive to inhaled methacholine and wheezed less frequently but coughed more frequently during methacholine-induced bronchoconstriction than did patients with classic asthma. Multivariate analysis revealed that airway hypersensitivity and lower baseline FEV_1_/FVC were associated with the appearance of wheezes, whereas a diagnosis of CVA was associated with coughing.

**Conclusion:**

There are mechanistic and phenotypic differences between CVA and classic asthma during methacholine inhalation. Frequent coughing during bronchoconstriction may be a distinctive feature of CVA.

## Background

Patients with cough variant asthma (CVA) present with a chronic cough as the sole symptom that responds to bronchodilator treatment and show airway hyperresponsiveness (AHR). CVA, one of the most common causes of chronic cough [1-4], is considered a precursor [5-9] and a variant form of classic asthma with typical symptoms of wheezing and dyspnea [5]. Several studies have examined mechanistic differences between CVA and classic asthma. Airway sensitivity, a component of airway responsiveness that is defined as the inflection point where respiratory resistance (Rrs) starts to increase, did not differ between patients with CVA and those with classic asthma in a few small studies [10,11]. In contrast, airway reactivity, another component of airway responsiveness expressed as the slope of the dose-response curve, is attenuated in children with CVA as compared with those with classic asthma [12]. In adults with CVA, however, no study has separately examined airway sensitivity and reactivity in a large number of patients.

Methacholine, a non-specific cholinergic stimulant, induces bronchoconstriction without exacerbating airway inflammation. Apart from an analysis of mechanistic aspects, analyses of phenotypes, such as the appearance of cough and wheezes during methacholine-induced bronchoconstriction, may provide clues to understanding the unique features of CVA. To our knowledge, however, such an approach has not been attempted thus far. In one study in asthmatic children, detection of wheezes during methacholine inhalation depended on the degree of airway narrowing, while factors related to coughing during methacholine inhalation were not specified [13].

In this study, we initially examined airway sensitivity and reactivity to methacholine in adults with CVA and in those with classic asthma, using a continuous inhalation method that can separately evaluate these two components [12,14]. Our major concern was the presence or absence of cough and wheezes during methacholine-induced bronchoconstriction. Factors associated with the appearance of cough and wheezes were then analyzed.

## Methods

### Study subjects and design

We cross-sectionally studied adults with classic asthma (n = 58) or with CVA (55) who presented at the outpatient clinic of Kyoto University Hospital from April 1993 to September 2001. Classic asthma was diagnosed according to the American Thoracic Society criteria [15]: the symptoms of episodic wheezing and dyspnea within the previous year that responds to bronchodilators, and AHR to methacholine inhalation.

CVA was diagnosed according to the following criteria [5,10]: an isolated chronic cough without wheezing or dyspnea that had persisted for more than 8 weeks, AHR to methacholine, and symptomatic improvement of coughing in response to inhaled beta-_2 _agonists, sustained-release theophylline, or both. Wheezing or rhonchi were not audible on chest auscultation, even with forced expiration. No patient had a past history of asthma or had an upper respiratory tract infection within the past 8 weeks. No other apparent causes of chronic cough, such as gastroesophageal reflux, chronic sinusitis, or medication with angiotensin-converting enzyme inhibitors, were present. Patients with CVA had normal chest radiographs and were steroid-naïve, similar to those with classic asthma. The ethics committee of our institution approved the study protocol, and written informed consent was obtained from each participant.

### Pulmonary function test and methacholine challenge test

Prebronchodilator FEV_1 _was tested using a spirometer (Chestac-65V, Chest, Tokyo, Japan) according to the standards of the American Thoracic Society [16].

Airway responsiveness was tested by directly recording a dose-response curve of Rrs (cmH_2_O/L/sec) during continuous inhalation of methacholine in two-fold incremental concentrations (49 to 25,000 μg/ml) under tidal breathing from nebulizers with an output of 0.15 ml/minute (Astograph™; Chest, Tokyo, Japan), as described previously in detail [14,17]. If bronchodilators were being used, their use was suspended 24 hours before the methacholine inhalation. In short, after we recorded the baseline Rrs during inhalation of physiologic saline for 1 minute, patients inhaled methacholine, starting with the lowest concentration, at 1-minute intervals. The index of airway sensitivity that we adopted was Dmin: the cumulative dose of inhaled methacholine at the inflection point where which Rrs began to increase continuously. One unit of Dmin is equivalent to dose of 1 mg/ml of methacholine inhalation for one minute. Inhalation of methacholine was discontinued, and switched to bronchodilator inhalation when Rrs reached twice the baseline value. The plateau of the dose-response curve was not, therefore, examined. The slope of the methacholine-Rrs dose-response curve (SRrs) was used as an index of airway reactivity. FEV1 was not measured after the methacholine challenge test since holding the administration of a bronchodilator and addition of forced expiratory maneuver might induce severe bronchoconstriction.

### Appearance of cough and wheezes during the methacholine challenge test

Cough was considered to have appeared during the methacholine inhalation when patients coughed one or more times after the inflection point of Dmin. Cough before the point of Dmin, if any, was also documented. Coughing caused a transient spike-shape increase in Rrs, but it did not interfere with the determination of the inflection point or the slope of the dose-response curve. When the methacholine inhalation was discontinued and switched to bronchodilator inhalation, whether wheezing was audible on auscultation was assessed by either of the attending physicians (AN, HM). The assessment of cough or wheezes was performed in a blinded manner.

### Capsaicin cough sensitivity test

In 18 patients with classic asthma and in 22 with CVA, cough sensitivity test in addition to methacholine inhalation test was done one to two weeks apart. Cough sensitivity was tested by a continuous inhalation method of capsaicin solution using the Astograph™ as described previously [18]. Ten doubling concentrations of capsaicin solution (0.61–312 μM) were inhaled until 5 or more coughs were induced (cough threshold, C5). Each concentration of capsaicin was inhaled for 15 seconds during tidal breathing every 60 seconds. Remaining patients were not examined for capsaicin cough sensitivity because informed consents for the test were not obtained mostly due to time constraint.

### Statistical analysis

Data were analyzed using GraphPad Prism 4.00 (GraphPad Software, Inc., La Jolla, CA, USA) and StatView software 5.0 (SAS Institute Inc, Cary, NC, USA). To compare the two patient groups, the t-test was used when data were normally distributed, and the Mann-Whitney test was used for nonparametric data. The χ^2 ^test was used for the comparisons of nominal data between groups. Univariate and stepwise multivariate regression analyses were performed to test for independent effects of disease diagnosis, blood eosinophil counts, atopic status, FEV1/FVC, current smoking, log Dmin, and SRrs levels on the appearance of cough or wheezes during methacholine inhalation, setting the absence of cough or wheezes as 0 and the presence as 1. We did not include C5 levels as an independent variable since less than half of the patients underwent the capsaicin cough sensitivity test. For inclusion of variables into multivariate analyses, the F value, a measure of the extent to which a variable makes a unique contribution to the prediction of the dependent variable, was set at 4.0. Data are expressed as means ± SD. We considered p values of < 0.05 to indicate statistical significance.

## Results

Patients' characteristics are shown in Table 1. As compared with classic asthma group, CVA group included fewer smokers, had a lower blood eosinophil count, a higher baseline FEV1/FVC value, a lower baseline Rrs value, and less sensitivity and less reactivity to inhaled methacholine.

**Table 1:** Patients' characteristics

	Classic asthma	Cough variant asthma	p-value
	n = 58	n = 55	
Age (yr)	44.4 ± 15.9	43.2 ± 16.5	0.71
Male/Female (No)	29/29	33/22	0.29
Disease duration at diagnosis (yr)	6.7 ± 10.0	2.8 ± 4.4	0.10
Current smoking (Yes, %)	19.0	5.2	0.029
Atopic status* (Yes, %)	71.4	67.9	0.69
Blood eosinophils (number/μl)	389 ± 247	310 ± 404	0.011
FEV_1 _(% predicted)	86.8 ± 19.2	92.1 ± 17.6	0.14
FEV_1_/FVC (%)	72.6 ± 11.3	81.8 ± 8.9	< 0.0001
Baseline Rrs (cm H_2_O/L/sec)	4.3 ± 2.0	4.0 ± 3.2	0.040
Log Dmin (units)	-0.20 ± 0.82	0.36 ± 0.60	< 0.0001
SRrs (cm H_2_O/L/sec/min)	2.9 ± 3.2	2.1 ± 2.1	0.042
Log C5 (μM)	1.51 ± 0.79 (n = 18)	1.17 ± 0.71 (n = 22)	0.15

Values are given as the means ± SD.

*: measured in 56 patients with classic asthma and 53 patients with cough variant asthma. Patients were considered atopic when 1 or more specific IgE antibodies were positive for cat dander, dog dander, weed, grass pollen, mold, or house dust mite.

Abbreviation: Rrs, respiratory resistance. Dmin, cumulative dose of inhaled methacholine at the inflection point, where which respiratory resistance begins to increase. SRrs, slope of the methacholine dose-response curve for Rrs. C5, cough threshold, the lowest concentration of capsaicin that induces 5 or more cough.

As for phenotypic characteristics during methacholine-induced bronchoconstriction, cough appeared in 19 patients (35%) in the CVA group and 10 (17%) in the classic asthma group (p = 0.035), whereas wheezes were detected at the end of inhalation in 9 patients (16%) in the CVA group and 28 (48%) in the classic asthma group (p = 0.0003). Four patients with CVA started to cough before the inflection point of Dmin. In three of these patients, cough was relieved when methacholine was switched to a bronchodilator, associated with a two-fold increase in Rrs from baseline. Multivariate analyses of the appearance of wheezes and cough showed that lower baseline FEV1/FVC and airway hypersensitivity were independently associated with the detection of wheezes (Table 2), whereas the appearance of cough was solely associated with a diagnosis of CVA (Table 3). These results were unchanged even when four patients with CVA who started to cough before the inflection point of Dmin were excluded from the analyses.

**Table 2:** Univariate and multivariate regression analysis of appearance of wheezes

	Univariate analysis Correlation coefficient	P	Wheezes Standardized partial regression coefficient	F value
Disease	0.34	0.0002	not entered	< 4.0
Blood eosinophils (number/μl)	0.11	0.35	not entered	< 4.0
Atopy	-0.05	0.60	not entered	< 4.0
Baseline FEV1/FVC (%)	-0.31	0.001	-0.31	9.2
Smoking	0.08	0.39	not entered	< 4.0
Log Dmin (units)	-0.30	0.001	-0.33	10.7
SRrs (cmH_2_O/L/sec/min)	0.28	0.003	not entered	< 4.0

Adjusted R^2 ^= 0.22, p < 0.0001 for the multivariate analysis of appearance of wheezes. Wheezes, atopy, and smoking status are rated as 0 for absent and 1 for present. Disease is labeled as 0 for cough variant asthma and 1 for classic asthma. F value is a measure of the extent to which a variable makes a unique contribution to the prediction of the dependent variable. Dmin, cumulative dose of inhaled methacholine at the inflection point, where which respiratory resistance begins to increase. SRrs, slope of the methacholine dose-response curve for respiratory resistance.

**Table 3:** Univariate and multivariate regression analyses of appearance of cough

	Univariate analysis Correlation coefficient	P	Cough Standardized partial regression coefficient	F value
Disease	-0.20	0.036	-0.27	6.2
Blood eosinophils (number/μl)	-0.12	0.27	Not entered	< 4.0
Atopy	0.02	0.83	not entered	< 4.0
Baseline FEV1/FVC (%)	0.11	0.25	not entered	< 4.0
Smoking	-0.10	0.30	not entered	< 4.0
Log Dmin (units)	0.09	0.33	not entered	< 4.0
SRrs (cmH_2_O/L/sec/min)	-0.02	0.87	not entered	< 4.0

Adjusted R^2 ^= 0.06, p = 0.015 for the multivariate analysis of appearance of cough. Cough, atopy, and smoking status are rated as 0 for absent and 1 for present. Disease is labeled as 0 for cough variant asthma and 1 for classic asthma. F value is a measure of the extent to which a variable makes a unique contribution to the prediction of the dependent variable. Dmin, cumulative dose of inhaled methacholine at the inflection point, where which respiratory resistance begins to increase. SRrs, slope of the methacholine dose-response curve for respiratory resistance.

Cough sensitivity did not differ between patients with classic asthma (n = 18) and those with CVA (n = 22) (Table 1). However, in CVA group 9 patients who coughed during the methacholine-induced bronchoconstriction showed more enhanced cough sensitivity to inhaled capsaicin (log C5, 0.72 ± 0.65 μM) than 13 non-coughers (1.48 ± 0.58 μM)(p = 0.015). Meanwhile, 2 coughers and 16 non-coughers in classic asthma group did not differ in their cough sensitivity (0.69 ± 0.85 μM; 1.62 ± 0.75 μM, respectively)(p = 0.12).

## Discussion

To our knowledge, this is the first study to comprehensively examine mechanistic and phenotypic differences during methacholine inhalation between adults with CVA and those with classic asthma. Patients with CVA were less sensitive and less reactive to inhaled methacholine than were those with classic asthma. Coughing was more frequent during methacholine-induced bronchoconstriction in the CVA group, whereas wheezes were more frequent in the classic asthma group at the end of methacholine inhalation. Multivariate analysis of factors related to cough and wheezes revealed that wheezes were associated with airway hypersensitivity and baseline airflow obstruction, whereas cough triggered by bronchoconstriction was related to CVA.

Airway sensitivity and reactivity are thought to be differently regulated [19,20]. Airway sensitivity is most likely associated with airway inflammation, epithelial damage or malfunction, abnormal neural control, and increased inflammatory cell number and activity. In contrast, airway reactivity is considered most strongly related to smooth muscle contractility. Airway sensitivity was substantially lower in patients with CVA than in those with classic asthma. Previous studies showed no significant difference in airway sensitivity between these two asthmatic conditions [10-12,21]. The discrepancy may be attributed to differences in patient selection [10-12,21] and methodology [21]. Children with CVA were studied by Koh *et al *[21] and Mochizuki *et al *[12]. The airway physiology of children may differ from that of adults, as suggested by differences between mature and immature animals in the responses of airway smooth muscle to cholinergic stimulation [22]. In previous studies of adults with CVA (n = 14) [10], (n = 10) [11], sample sizes were relatively small. In addition, our previous study was conducted in patients with CVA who agreed to be hospitalized and to undergo bronchoscopic examination [10], conditions that might have lead to a selection bias toward patients with more severe CVA. In contrast, all subjects with CVA in the present study were outpatients. Our subjects may therefore be more representative of patients encountered in daily practice.

Several studies in children indicate that the degree of excessive airway narrowing is modest in patients with CVA. Yoo *et al *have shown that children with CVA more frequently reach a maximal response plateau on the dose-response curve to methacholine than those with classic asthma [23]. Moreover, plateau levels are lower in children with CVA [24]. In agreement with these results in children with CVA [12,23,24], we demonstrated for the first time that adults with CVA were significantly less reactive to methacholine than were those with classic asthma, although the difference in airway reactivity between the two groups was small in our study of adults.

The presence or absence of cough and wheezes during methacholine-induced bronchoconstriction was our main interest. Bronchoconstriction is a well-known stimulant of cough that is thought to be mediated by mechanosensitive, rapidly adapting receptors [25]. In a guinea pig model of CVA, degree of antigen-induced bronchoconstriction is strongly correlated with cough counts that are inhibited by procaterol administration [26]. Clinical studies examining the appearance of cough during bronchoconstriction are scant, however. Springer *et al*. performed methacholine provocation tests by the forced expiration method in asthmatic children with wheezing [13]. Cough appeared in most (81%) of the asthmatic children, but the background characteristics of the coughers were not described. We showed that the appearance of cough was solely associated with a diagnosis of CVA and not with mechanistic variables. It was also surprising that only 17% of the adults with classic asthma coughed during bronchoconstriction in our study. Mechanisms underlying the discrepancy in bronchoconstriction-induced cough between classic asthma and CVA were not clarified since cough sensitivity did not differ between the two asthmatic conditions. However given that in CVA group coughers had more heightened cough sensitivity to inhaled capsaicin than non-coughers, cough during methacholine-induced bronchoconstriction might be on a background of enhanced capsaicin cough reflex in CVA. However the guinea pig model of CVA described above is not sensitive to inhaled capsaicin and the authors negate the involvement of tachykinins in bronchoconstriction-induced cough [26]. Further studies are necessary to elucidate a linkage between bronchoconstriction-triggered cough and capsaicin-induced cough in CVA patients.

As expected, wheezes were more frequent in the classic asthma group than in the CVA group at the end of methacholine inhalation. In contrast to cough, wheezes were not classic asthma-specific on multivariate analysis. Baseline airflow obstruction and airway hypersensitivity contributed to the presence of wheezes, consistent with the theory that wheezes are generated by airflow turbulence [27]. Mochizuki *et al*. proposed that lower airway reactivity or slower airway constriction may explain the absence of wheezing or dyspnea in children with CVA [12]. We found no independent contribution of airway hyperreactivity to the appearance of wheezes. The lower frequency of wheezes in patients with CVA may be inherently related to their better pulmonary function and modest airway sensitivity.

Needless to say, airway inflammation has an important role in the pathogenesis of both CVA and classic asthma. Lack of the information on airway inflammation in the present study may not weaken our results, however, since methacholine contracts airway smooth muscle without modulating airway inflammation. Methacholine provocation test may not reproduce clinical conditions, but we believe that our findings regarding the frequency of cough and wheezes triggered by airway smooth muscle contraction in classic asthma and CVA are novel and relevant. One may argue that methacholine worked as a non-specific nociceptor stimulant for cough. However, given that cough subsided after methacholine was switched to a bronchodilator, we are convinced that cough was triggered directly by bronchoconstriction. Another possible limitation of this study was that wheezes were not automatically detected. Albeit auscultation is less sensitive than automatic analysis, their agreement is fairly good [27], and auscultation was done in a constant and blinded manner by either of the two examiners (HM, AN) to minimize bias.

## Conclusion

In conclusion, there are mechanistic and phenotypic differences between CVA and classic asthma during methacholine-induced bronchoconstriction. The milder mechanistic impairment in patients with CVA may explain their lower frequency of wheezing. Frequent coughing triggered by bronchoconstriction was predominantly associated with CVA and was unrelated to mechanistic variables. Our findings may provide important clues to better understanding the unique features of CVA.

## Competing interests

The authors declare that they have no competing interests.

## Authors' contributions

H Matsumoto conceived the whole study, contributed to its design, acquisition and interpretation of data, and drafted the manuscript. AN conceived the study, contributed to its design, data acquisition, and data interpretation. MT participated in acquisition of data. TU participated in acquisition of data. MY participated in acquisition of data. HM participated in acquisition of data. MJ participated in acquisition of data. KC contributed to data interpretation. MM contributed to data interpretation
